# Nitrogen Accumulation in Forests. Exposure Monitoring by Mosses

**DOI:** 10.1100/tsw.2007.11

**Published:** 2007-03-21

**Authors:** Roland Pesch, Winfried Schröder, Gunther Schmidt

**Affiliations:** Department of Landscape Ecology, University of Vechta, PO 1553, D-49356 Vechta, Germany

**Keywords:** biomonitoring, data compilation, geostatistics, mosses, nitrogen accumulation

## Abstract

At present, there is still little information on nitrogen (N) accumulation in forests contrasting with the crucial importance of N in forest ecosystems. This work analyzes the N bioaccumulation in mosses from forested areas from Lower Saxony and North Rhine-Westphalia (two of 16 federal states of Germany), the Weser Ems Region (part of Lower Saxony), and the Euro Region Nissa (covering the Czech Republic, Germany, Poland). The studies involved samples collected from 190 sites between 1998 and 2005. Different spatial scales and regional differences in land use were chosen to assess the factors affecting N bioaccumulation in forested areas. A continuous reduction of N bioaccumulation was found from Lower Saxony (a region where agriculture is most predominant) to North Rhine-Westphalia (mostly urban). The Weser Ems Region (an agricultural region) showed a higher N concentration in mosses than the Euroregion Nissa (a former industrial region). Statistical analyses performed at the different spatial scales revealed that the areas showing greater agricultural and livestock spatial densities favor N bioaccumulation in mosses. N concentration in mosses was moderately correlated with the N concentration in the leaves and needles of the surrounding trees. No significant relationships were found regarding the crown density of forest trees or N deposition estimations from a combination of atmospheric models and deposition measurements.

## GOALS AND BACKGROUND

Temperature, radiation, water, and nutrient supply are the main abiotic controls of primary production. Atmospheric depositions of nitrogen (N) are likely to enhance growth for many temperate coniferous forests sites where N is considered to be the most common limiting nutrient. However, a saturation of N may occur correlated with the leaching of nutrients such as Ca, Mg, and P as well as an increase of C sequestration. Exposure to N-containing air pollutants influences the subsequent response of plants to drought, frost stress, and parasite attack. There is also evidence that environmental conditions like habitat or air temperature can affect the response of plants to N exposure[1,2,3].

Although the emissions of N oxides in Europe have been reduced by 25% from 1980–2000, large regional differences in N emissions have been recorded and, therefore, N deposition is still exceeding the critical loads in large areas throughout Europe. About half of ICP Waters sites exhibit a high degree of N saturation and N deposition at 92% of 230 ICP Forests level II plots, such that N would continue to accumulate in the soils. Thus, those ecosystems are expected to reach N saturation sooner or later[4]. The 1999 Gothenburg Protocol should help to slow down the process, but N will continue to accumulate in terrestrial ecosystems, thus increasing the risk of the long-term saturation.

Because of the ecological significance of N for ecosystems, broad-scale programs to monitor N deposition on forests are needed. Mosses have been proposed to determine spatial patterns of N deposition[5], providing a time-integrated measure of the spatial bioaccumulation pattern. In fact, a clear relationship between atmospheric N deposition and the concentration of N in mosses has been found[6,7]. Therefore, mosses have been recently used in a pan-European assessment of N deposition[8]. Significant differences between countries were recorded. For instance, the total N concentrations in mosses from Finland, Norway, and Sweden were about 1%, while the values from Germany were around 2%. These differences in N accumulation might be explained by land-use patterns, but a monitoring program with greater spatial resolution is needed to test this hypothesis.

Therefore, we performed a study to assess and explain the spatial patterns of variation in N concentrations in mosses found throughout the German federal states of Lower Saxony and North Rhine-Westphalia, and the Weser-Ems Region (WER), both being part of Lower Saxony, and the Euro Region Nissa (ERN), respectively. The sampling spatial resolution involved in this study and the great differences in land use found in those regions provided an excellent scenario to define the potential relationships between N concentration in mosses and land use patterns.

## MATERIAL AND METHODS

### Study Areas

The study areas (Fig. 1) were selected due to differences in actual and historical land use: Lower Saxony is dominated by agricultural land with highest densities in the WER, while North Rhine-Westphalia encompasses the largest urban agglomeration in Europe (Table 1). The ERN was an industrial center covering the former German Democratic Republic, the Czech Republic, and Poland until 1989. Accordingly, the regions should differ with regard to the emissions of different atmospheric pollutants. Unfortunately, no spatially disaggregated data on emission sources are available. Emissions from urban and industrial regions are likely to be dominated by metals, while agricultural areas would be dominated by N emissions

### Monitoring the N-Accumulation in Mosses

Moss sampling was performed during 1998–2001 at 133 sites in Lower Saxony and North Rhine-Westphalia; 28 and 24 sites were sampled in the WER and ERN region, respectively. Sampling followed a standard protocol that was applied in pan-European moss surveys carried out in 1990, 1995, 2000, and 2005[10]. Accordingly, the mosses were sampled at least 100 m afar from any ordinary road or single house; 300 m from settlements, primary roads, and highways; and 1000 m from industries, respectively. In North Rhine-Westphalia, samples of three moss species, *Pleurozium schreberi (P.s.), Scleropodium purum (S.p.),* and *Hypnum cupressiforme (H.c.,),* were collected at 81 sites (autumn/winter 2000/2001) and in Lower Saxony at 42 sites (autumn/winter 1998/1999). In the WER, the same species were sampled at 28 sites (December 2004) and 24 sites were sampled in the ERN (March 2005). In each of the moss samples, N concentration was determined (in percent dry weight). To control and ensure the quality and comparability of the measurements, the standard reference material from the European moss surveys was used[11]. Each of the sampling sites was described with regard to those local boundary conditions that might influence N accumulation in mosses. The respective site-specific information was documented in a

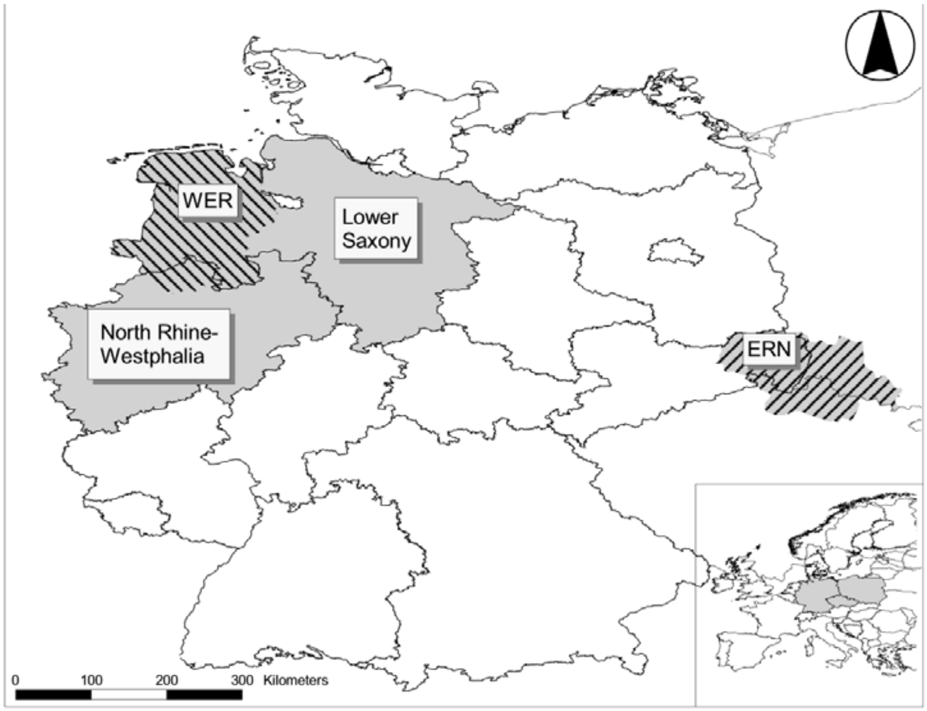

**FIGURE 1.** Geographical location of the regions involved in the study.

**TABLE 1**
**Land Use in Europe (EU), Germany, North Rhine-Westphalia (NRW), Lower Saxony (LS), WER and ERN (Percentages of Areas, Calculated From Corine Landcover Data 2000[9])**

| | EU | Germany | LS | NRW | WER | ERN |
|---|---|---|---|---|---|---|
| Urban | 4 | 8 | 7.3 | 11.5 | 6 | 7 |
| Farmland | 36.3 | 45.8 | 52.1 | 48.3 | 46 | 40 |
| Pastures, Meadows | 11 | 13 | 15.2 | 11.3 | 29 | 11 |
| Forests | 35.8 | 29.4 | 20.5 | 27.6 | 9 | 40 |
| Moor | 2 | 0.4 | 0.9 | 0.4 | 2 | 0 |
| Other | 10.9 | 3.5 | 4 | 0.8 | 8 | 2 |

database, including coordinates, date of sampling, administrative district, name of moss sampler, moss species, number of subsamples (sampling points), altitude, inclination and aspect, vegetation-related parameters aspects (distance between the sampling points and trees, height of surrounding trees, dominant tree species, low canopy, and herbaceous layer), as well as land use–related parameters (agricultural land coverage, distances to roads, human settlements, and industrial facilities).

## Data Integration and Analysis

The data sets on N accumulation and site-specific characteristics (land use 2000[9], N deposition derived by combination of deposition models and data for 1999[12], geostatistical estimations on crown density of forest trees observed in 2000[13], and on N in leaves and needles measured in 2000[14]) were integrated in a geographic information system (GIS) using ArcView GIS 3.3. These data were evaluated by means of descriptive statistics (box plots), correlation and regression analysis, Classification and Regresion Trees (CART), and geostatistics.

The kriging interpolation technique was used to perform data spatial interpolation. Data were previously tested for spatial autocorrelation before the kriging interpolation was carried out. The accuracy of the exercise was assessed through cross-validation analysis, by sequentially extracting the data from each measurement site and estimating it according to the semi-variogram model. The differences between the measured and geostatistically estimated values were described by suitable statistical measures[15].

### Classification and Regression Trees (CART)

In this study, CART[16] was used to calculate the correlations between the N bioaccumulation and characteristics of the sampling sites that could locally modify the accumulation values. CART does not make any assumptions regarding the distribution of the data and can use an explanatory variable more than once, so it can work with multiple interrelated data. CART can reveal hierarchical and nonlinear relationships among one dependent variable (N bioaccumulation) and several describing variables (sampling sites characteristics) by subdividing a heterogeneous data set into more homogeneous subsets (classes, groups, nodes) by a series of nested binary “if-then-else” splits. Each split maximizes the homogeneity of the dependent variable. Each possible binary split for all variables is evaluated recursively for the best class separation until homogeneous end points (nodes) are reached. The predictor selected is the one for which the two new classes have the greatest within-group similarity for the response variable. The two new classes are then examined separately with respect to each of the predictor variables to see if they can be split again. The resulting dendrogram can have multiple branches, each of which represents a path to a particular combination of independent variables defining variable subspaces.

## RESULTS AND DISCUSSION

### Estimation of the Regional Patterns of N Bioaccumulation

To assess the N bioaccumulation within forests in Lower Saxony[17] and North Rhine-Westphalia[18], the N concentrations in mosses measured at 133 sites were analyzed geostatistically (Fig. 2). The resulting variogram model depicts a weak, but distinct, autocorrelation structure of the measured values with a nugget/sill ratio of 50% and a range of 140 km. Ordinary kriging was applied to calculate a raster map with a resolution of 1,000 m × 1,000 m. The resulting map illustrates a continuous decrease of N bioaccumulation from Lower Saxony to North Rhine-Westphalia with highest values of above 2.2% N in the WER and lowest values in the south of North Rhine-Westphalia (<1.4% N). This gradient appears to follow a decreasing coverage of agricultural areas. From the results of the cross-validation, a mean percentage error of 11.6% was calculated. Subsequently, the estimated N map was intersected with a vector map on forest areas derived from the Corine landcover map and a 100 m × 100 m raster map on the percentage of agricultural land use within a 5-km buffer around the moss sampling sites, here referred to as agriculture index (Fig. 2). For all forests within the study area, a mean bioaccumulation of 1.9% N was estimated with a minimum of 1.3 and a maximum of 2.3% N. The areas exhibiting agriculture indices of less than 50% showed a lower N accumulation (median = 1.69% N) than those forested areas exhibiting indices of above 50% (median = 1.93%). For the latter areas the interquartile range lies between 1.55–1.97% N.

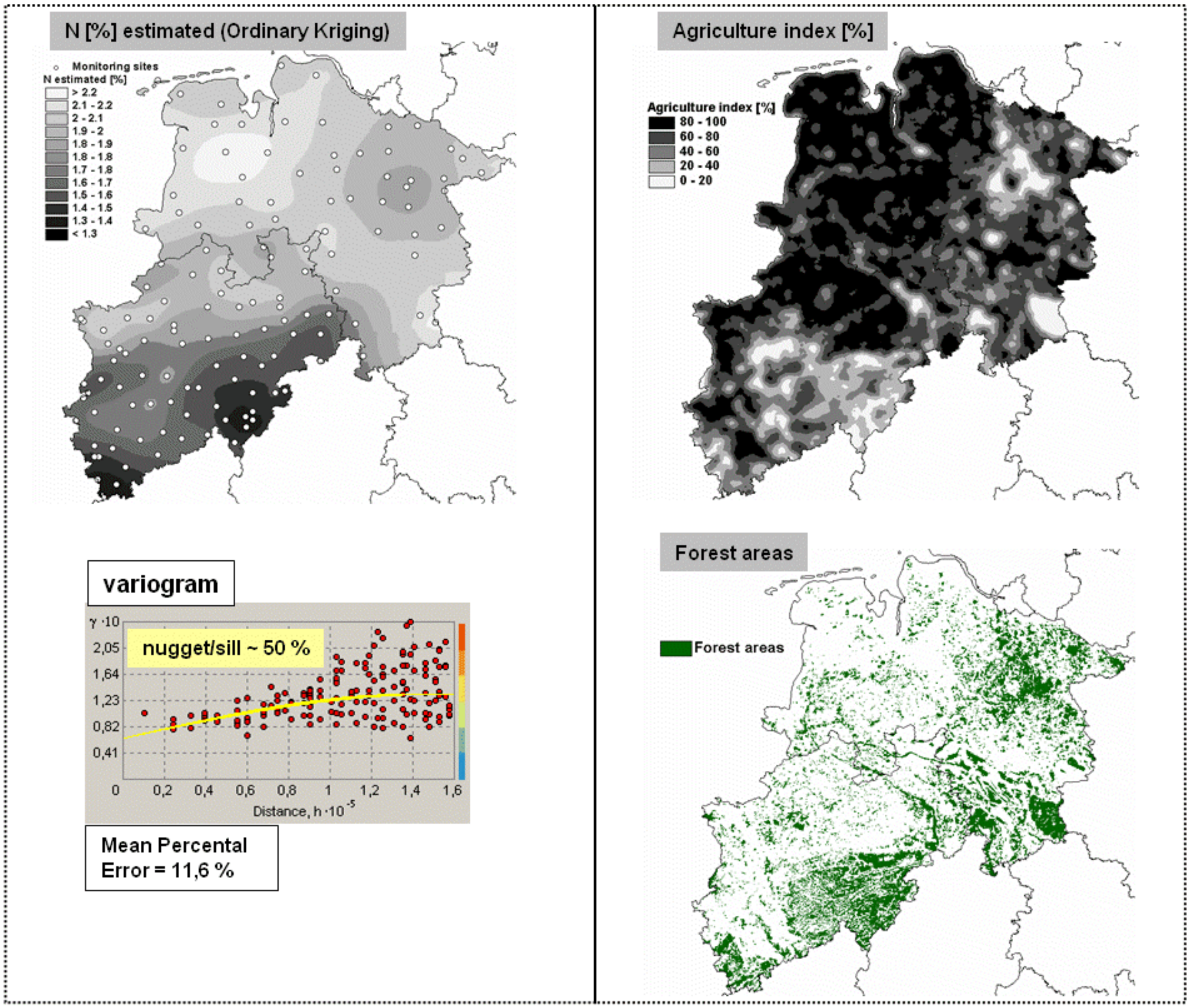

**FIGURE 2.** Estimated N bioaccumulation in mosses, agriculture indices, and forest areas in Lower Saxony and North Rhine-Westphalia.

With regard to the comparison between the ERN and the WER, it can be stated that the estimated N bioaccumulation within forests in the WER exceeds that of the ERN by almost 1%. Whereas in the WER a median of 2.2% could be calculated from the estimated N loads, the ERN shows a median of 1.4% N.

## Sampling Site-Specific Variation of the N Accumulation in Mosses from the ERN and WER

To assess whether the measured N bioaccumulation rates in mosses could be affected by site characteristics other than N deposition, CART was applied. Due to restrictions related with the availability of data, only data from WER and ERN were included in this analysis. The N bioaccumulation measured in both regions was set as the target variable. The elevation; the distance of the sampling site to the nearest trees, to bushes, to roads, to settlements, and to industries; the weather conditions during sampling; and the moss species were defined as the predictor variables. The percentage of agricultural land use within a 5-km buffer around the monitoring site was chosen as an additional predictor to account for the N agricultural emissions. As can be seen in Fig. 3, the root node is at first split by the elevation

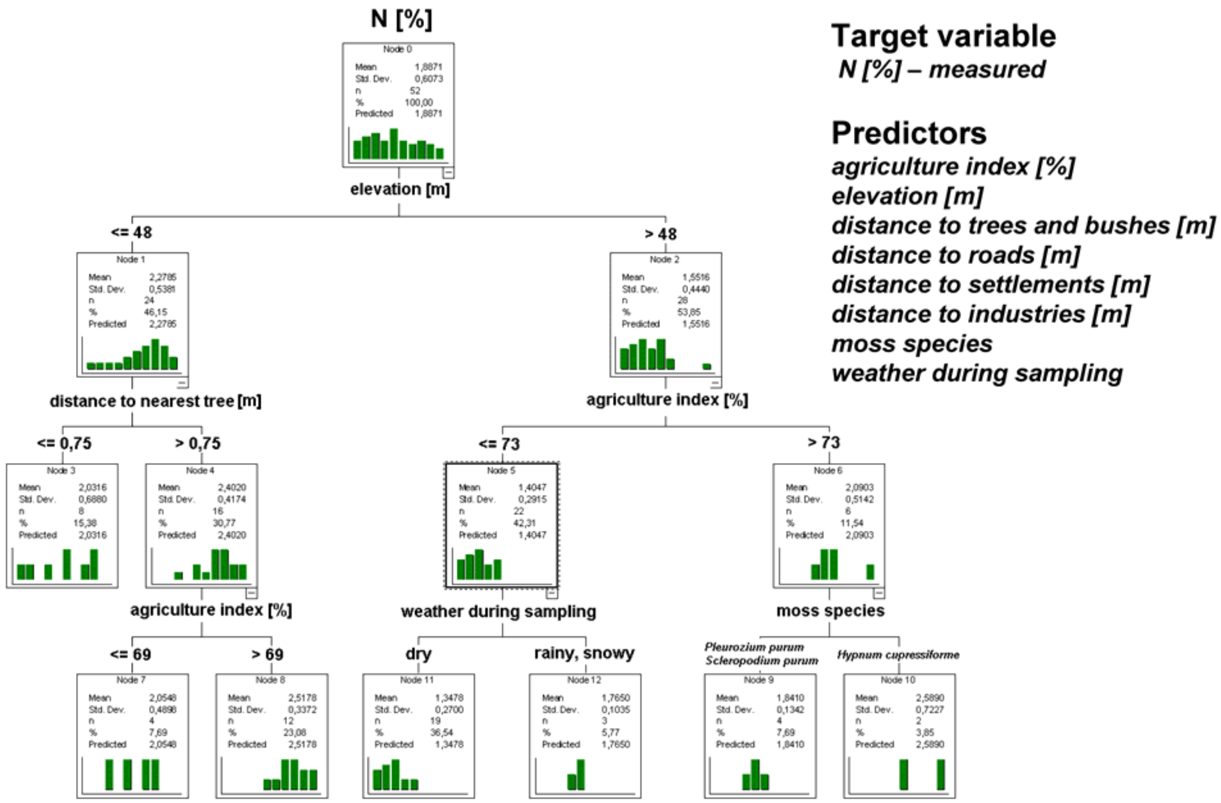

**FIGURE 3**. Site-specific variation of N accumulations in mosses from the ERN and WER, 2005.

at 48 m, subdividing the data set according to the two compared regions (node 1: WER; node 2: ERN). Node 1 is then split by the distance of the sampling site to the nearest tree. Sites sampled within less than 0.75 m afar from trees show slightly lower N accumulation (mean = 2% N) than those sampled more than 0.75 m from trees (mean = 2.4% N). These nodes were subsequently subdivided by the agriculture index into end-nodes 7 and 8: Sites showing less than 69% agricultural land-use coverage in the surrounding areas exhibit lower N accumulation in the mosses than sites with more than 69% (mean = 2 and mean = 2.5% N, respectively). The same tendency can be observed with regard to node 2 that stands for the ERN splitting the data set into node 5 (agriculture index ≤73%) and node 6 (>73%). Furthermore, the moss species are chosen to split node 6 into one end-node containing sites where *Hypnum cupressiforme* and one node where *Pleurozium schreberi* and *Scleropodium purum* were sampled. The node containing *H. cupressiforme* shows higher N accumulation (2.6% N). Node 5 is furthermore subdivided by the predictor “weather during sampling”. Sites sampled during rainy and snowy weather show higher N values (mean = 1.77% N).

## Relationships Between N Accumulations in Mosses and Land Use, N Deposition, Leaf N Concentrations, and Crown Density of Forest Trees

Taking into account the above-described site-specific variation of the N accumulation in mosses, the relationships between N accumulations in mosses and the spatial density of agricultural land use and livestock, N deposition, leaf N concentrations, and crown density of forest trees were analyzed by means of correlation coefficients (Pearson, Spearman) and regression equations. Some of the results are documented in Table 2.

**TABLE 2**
**Statistical Relations Between N Accumulations in Mosses and Land Use, Modeled N Deposition, Crown Density, and N Concentrations in Leaves and Needles of Forest Trees**

| N in Mosses* correlated with ... | r (Pearson) | | r (Spearman) | | Regression | $R^2$ |
|---|---|---|---|---|---|---|
| | r | p | r | p | | |
| Spatial density of agricultural land use in LS and NRW | 0.35 | < 0.001 | 0.41 | < 0.001 | y = 22.143 x + 17.462 | 0.12 |
| Spatial density of agricultural land use in NRW | 0.39 | < 0.001 | 0.38 | < 0.001 | y = 22.183 x + 16.897 | 0.15 |
| Spatial density of agricultural land use in ERN and WER | 0.54 | < 0.001 | 0.52 | < 0.001 | y = 21.141 x + 22.685 | 0.29 |
| Spatial density of agricultural land use in ERN | 0.39 | 0,062 | 0.37 | 0,075 | y = 21.196 x + 15.009 | 0.15 |
| Spatial density of livestock in LS and NRW | 0.32 | 0,010 | 0.32 | 0,007 | y = 60355 x - 31612 | 0.11 |
| Spatial density of livestock in ERN and WER | 0.51 | 0,005 | 0.56 | 0,001 | y = 86493 x - 58578 | 0.26 |
| N in leaves of forest trees NRW | 0.33 | 0,003 | 0.34 | 0,002 | y = 1982 x + 23753 | 0.11 |
| N in needles of forest trees NRW | 0.39 | < 0.001 | 0.34 | < 0.001 | y = 1744 x + 13773 | 0.16 |
| crown density NRW | 0,08 | 0,510 | 0,11 | 0,321 | y = 1.5121x + 17.314 | 0,006 |
| N-deposition in LS and NRW | -0,06 | 0,644 | -0,04 | 0,438 | y = -110.93x + 2987.4 | 0,002 |
| N-deposition in ERN and WER | 0,08 | 0,647 | 0,13 | 0,486 | y = 140.73x + 2534.8 | 0,006 |

*ERN = Euro Region Nissa (covers part of the Czech Republic, Germany, Poland)*
*WER = Weser-Ems Region (Northwestern Part of Lower Saxony)*
*LS = Lower Saxony NRW = North Rhine-Westfalia*

The results of the correlation and regression analysis show moderate statistical relationships between N concentrations in mosses and the spatial density of agricultural land use and livestock, and N concentrations in forest trees. The explained variance ($R^2$) ranged between 11 and 29%. No statistical relationships were found regarding tree crown density or N deposition[12]. The lack of correlation between N deposition and N in mosses might be due to the different methodological approaches. The N deposition map was calculated using deposition models combined with measurement data from different German deposition networks providing data from open-field and throughfall measurements. With regard to the measured N loads in the mosses, samples were only available from 133 or 52 sites, respectively. Here, the mosses were exclusively sampled within the influence of canopy drip.

Therefore, it can be concluded that high accumulation of N in mosses correlates with high agricultural land-use density in the vicinity of the forests. To improve knowledge on mechanisms and N impacts (e.g., eutrophication) in forest ecosystems, these findings should be corroborated by long-term investigations on spatial patterns and temporal trends on N bioaccumulation. Additionally, N deposition measurements and studies on biogeochemical fluxes should be carried out at the forested areas where N bioaccumulation is measured. Currently, N in mosses is monitored over large areas all over Europe. To ensure the synergy of monitoring activities, the moss monitoring network was linked with 88 sites of the ICP forests Level II network and three sites of the ICP Integrated Monitoring[19]. Thus, future studies are envisaged to establish at the country level the relationships between N concentrations in mosses and N deposition measurements, N concentrations in needles and leaves, the crown density of the trees, and the species composition of the forest vegetation. This will enable to further document the responses of forests to the combination of different environmental factors and the N input the forests are exposed to.
